# Radiographic Detection of Post-Traumatic Bone Fractures: Contribution of Artificial Intelligence Software to the Analysis of Senior and Junior Radiologists

**DOI:** 10.5334/jbsr.3574

**Published:** 2024-04-25

**Authors:** Andrea Dell’Aria, Denis Tack, Najat Saddiki, Sonia Makdoud, Jean Alexiou, François-Xavier De Hemptinne, Ivan Berkenbaum, Carine Neugroschl, Nunzia Tacelli

**Affiliations:** 1 Department of Radiology, Hôpitaux Iris-Sud (HIS), Université libre de Bruxelles (ULB), Brussels, Belgium; 2 Department of Radiology, Centre Hospitalier EpiCURA, Université libre de Bruxelles (ULB), Brussels, Belgium; 3 Department of Radiology, Hôpitaux Iris-Sud (HIS), Université libre de Bruxelles (ULB), Brussels, Belgium; 4 Department of Radiology, Hôpitaux Iris-Sud (HIS), Université d’Alger 1- Faculté de Médecine d’Alger-Ziania, Algiers, Algeria; 5 Department of Radiology, Hôpitaux Iris-Sud (HIS), Université libre de Bruxelles (ULB), Brussels, Belgium; 6 Department of Radiology, Hôpitaux Iris-Sud (HIS), Université libre de Bruxelles (ULB), Brussels, Belgium; 7 Department of Orthopaedic Surgery, Hôpitaux Iris-Sud (HIS), Université libre de Bruxelles (ULB), Brussels, Belgium; 8 Department of Radiology, Hôpitaux Iris-Sud (HIS), Université libre de Bruxelles (ULB), Brussels, Belgium; 9 Department of Radiology, Hôpitaux Iris-Sud (HIS), Université libre de Bruxelles (ULB), Brussels, Belgium

**Keywords:** Artificial intelligence, musculoskeletal, fracture, CBCT, diagnosis

## Abstract

**Objectives:**

The aims of this study were: (a) to evaluate the performance of an artificial intelligence (AI) software package (Boneview Trauma, Gleamer) for the detection of post-traumatic bone fractures in radiography as a standalone; (b) used by two radiologists (osteoarticular senior and junior); and (c) to determine to whom AI would be most helpful.

**Materials and Methods:**

Within 14 days of a trauma, 101 consecutive patients underwent radiographic examination of the upper or lower limbs. The definite diagnosis for identifying fractures was: (a) radio-clinical consensus between the radiologist on-call who analyzed the images and the orthopedist (Group 1); (b) Cone Beam computed tomography (CBCT) exploration of the area of interest, in case of doubts or absence of consensus (Group 2). Independently of this diagnosis for both groups, the radiographic images were separately analyzed by two radiologists (osteoarticular senior: SR; junior: JR) prior without, and thereafter with the results of AI.

**Results:**

AI performed better than the radiologists in detecting common fractures (Group 1), but not subtle fractures (Group 2). In association with AI, both radiologists increased their overall performances in both groups, whereas this increase was significantly higher for the JR (*p* < 0.05).

**Conclusion:**

AI is reliable for common radiographic fracture identification and is a useful learning tool for radiologists in training. However, the software’s overall performance does not exceed that of an osteoarticular senior radiologist, particularly in case of subtle lesions.

## Introduction

Artificial intelligence (AI) is playing an increasingly important role in the medical field, particularly in radiology. This new tool is used, among other things, to increase the detection rate of bone fractures on radiographic images, a frequent reason for visiting the medical imaging department, and a frequent cause of misdiagnosis [1]. Indeed, AI increases the sensitivity in detecting common fractures (distal radius, proximal femur, ankle, proximal humerus, and metacarpal fractures), which are usually easily detected by radiologists [2–14]. Nowadays, there are already several approved (Medical Device Regulation Certification) software packages of this type on the market, such as Boneview Trauma (*Gleamer*) [8], a deep learning AI using a deep convolutional neural network (DCNN) based on ‘detectron2,’ a generic object detection framework. It has been shown to provide excellent results in detecting common bone fractures of the extremities in both adults and children [9–11]. Its overall performance is similar to that of a musculoskeletal (MSK) radiologist and with AI, they can improve sensitivity by 6.5%–8.7% and specificity by 2.8%–4.1% [8, 12, 13].

However, there is little data on the sensitivity of AI in more complex situations, as may be the case for subtle fractures (fractures more frequently overlooked on initial radiographs) [15], and also on its usefulness to increase JR’s performances. Though, a peak in bone fracture detection error has been observed between 8 p.m. and 2 a.m., a time slot during which radiologists may be on call and unable to examine patients themselves [16].

It is yet unclear whether AI really has the potential to become a perfect substitute for experienced radiologists in the detection of all types of fractures or not, and whether AI should only be considered as a learning tool for less experienced radiologists, such as JR in training.

The aim of this study is therefore, first, to assess whether the performance of AI software surpasses that of radiologists, regardless of their experience, in the radiographic detection of post-traumatic common and subtle bone fractures, and second, to determine who can most benefit from its use.

## Materials and Methods

This prospective study has been approved by the Ethics Committees and written informed consent was obtained from all patients.

### Subject recruitment

During daytime clinical practice (from March 2, 2023 to March 30, 2023), a population of 101 consecutive patients was prospectively recruited on the basis of the following inclusion criteria: adults (>18 years) who had suffered a recent (<14 days) low-velocity trauma to the upper or lower limbs (including shoulders and hips) at a specific point (polytrauma excluded) for whom a doctor suspected a bone fracture. For the 101 patients, this was the first imaging investigation following their trauma. Patients who had undergone radiography between 5.30 p.m. and 8.30 a.m. were excluded from the study.

### Performing examinations

The 101 patients underwent radiography, with multiple incidences depending on the area of interest, according to the written procedures available in the department. The images were produced using the same two radiographic devices with identical acquisition parameters on the flat panel sensor.

### Image analysis

In clinical practice, the images were analyzed by the radiologist in charge of radiography (>15 years’ experience), who took no part in the reading sessions. This radiologist had access to all the medical information needed to interpret the images and was able to examine the patient, if necessary. He then reported the different lesions detected on the radiographic images. On the basis of a discussion with the orthopaedic surgeons, this radiologist had to decide if a cone beam computed tomography (CBCT) examination was needed. This happened within a maximum of 2 hours after the radiography had been obtained, mostly in case of radio-clinical doubt. The CBCT examination was analyzed by the same radiologist.

Radiographic images were sent to the Picture Archiving and Communicating system (PACS) and to a dedicated computer for the AI software (Boneview Trauma, Gleamer) analysis. AI processed the images in a few minutes and sent its results (positive, negative, and doubtful) to a particular database of the PACS.

During a first reading session, two radiologists, SR (>15 years’ experience) and JR (<5 years’ experience), reviewed the radiographic images independently. They assessed the presence or absence of a recent fracture on the images without knowledge of any other clinical information and had to express their response as positive, negative, or doubtful. They were also asked to locate positive or doubtful results.

Three months later, during a second reading session, the two readers were asked to read these examinations again and were asked to use AI results this time for the analysis.

### Group formation and statistical methods

The patients were divided into two groups according to the method of reference used:

- Group 1 consisted of patients for whom a clear radio-clinical consensus had been reached between the radiologist in charge of radiography and the orthopaedic surgeon. If the consensus was in favor of a diagnosis of fracture, the patients benefited from orthopaedic and radiological follow-up, yielding to a later demonstration of consolidation patterns that furthermore confirmed the positive diagnosis.
  In case of a consensus for the absence of any fracture, a follow-up with a consultation was carried out remotely to ensure this result.
- Group 2 was made up of patients for whom a clear diagnosis could not be reached in consensus, including fractures difficult to assess definitely and/or radio-clinical discrepancies for doubts. Patients in this group benefited from a CBCT examination, providing high-resolution 3D reformat images at a radiation dose lower than that of a computed tomography (CT) examination [17, 18]. The CBCT images were read by the radiologist in charge of the radiography and served as method of reference.

Statistical analyses included the following: sensibility (Se), specificity (Sp), are under the curve (AUC), odds ratio (OR) with a measure of 95% confidence interval (95% CI), positive predictive value (PPV), and negative predictive value (NPV).

The Wald test was used to determine the association between the reader’s results and the method of reference. *P* values lower than 0.05 indicated statistical significance.

During this study, we considered multiple fractures when more than one fracture was seen in more than one bone segment. In this case, the positive response of AI was considered valid even if it concerned only one fracture segment.

Doubtful responses were considered positive results because, in clinical practice, they result in the same treatment.

## Results

Our study population consisted of 50 men and 51 women, with a median age of 39 years.

A definite diagnosis of fracture was made in 54/101 patients (53.9%) on the basis of the consensus between the radiologist on-call and the orthopaedic surgeon for Group 1 and the CBCT results supplied by the radiologist in charge for Group 2. Fractures were of the following types: simple (59.2%), avulsion/tear-off (20.4%), multiple (14.8%), and comminuted (5.5%).

The distribution of the location of the radiographic images taken during the study between the two groups is shown in Table 1. CBCT examinations were positive for 32 out of 51 patients (64%) in Group 2.

**Table 1:** Distribution of anatomical regions explored by radiography.

LOCATION	GROUP 1	GROUP 2	TOTAL
**SHOULDER**	1	2	3
**ELBOW/FOREARM**	2	2	4
**WRIST**	7	22	29
**HAND**	12	9	21
**KNEE/LEG**	2	0	2
**ANKLE**	14	9	23
**FOOT**	12	7	19
**TOTAL**	50	51	101

Readers’ performance is illustrated in Tables 2 and 3 for Group 1, and in Tables 4 and 5 for Group 2. Follow-up didn’t show any missed fractures made by the radiologist in charge during the reading of radiographs and CBCT. All fractures were present on the initial images.

**Table 2:** Diagnostic performance of the two readers and AI in Group 1 during the first reading.

	SE (%)	SP (%)	AUC	*P*-VALUE	OR [95% CI]	PPV (%)	NPV (%)
**SR**	86.36	89.29	0.8782	<0.0001	53.0 [9.6–291.2]	86.36	89.29
**JR**	50.00	82.14	0.6607	0.0193	4.6 [1.3–16.5]	68.75	67.65
**AI**	95.45	89.29	0.9237	<0.0001	175.0 [16.0– > 999.9]	96.15	87.50

**Table 3:** Diagnostic performance of the two radiologists using AI in Group 1 during the second reading.

	SE (%)	SP (%)	AUC	*P*-VALUE	OR [95% CI]	PPV (%)	NPV (%)
**SR + AI**	95.45	89.29	0.9237	<0.0001	175.0 [17.0– > 999.9]	87.50	96.15
**JR + AI**	77.27	89.29	0.8328	<0.0001	28.33 [6.0–134.6]	85.00	83.33

**Table 4:** Diagnostic performance of the two readers and AI in Group 2 during the first reading.

	SE (%)	SP (%)	AUC	*P*-VALUE	OR [95% CI]	PPV (%)	NPV (%)
**SR**	56.25	89.47	0.7286	0.0039	10.9 [2.2–55.4]	90.00	54.84
**JR**	31.25	89.47	0.6036	0.1073	3.9 [0.7–20.0]	83.33	43.59
**AI**	68.75	36.84	0.5280	0.6824	1.3 [0.4–4.2]	64.71	41.18

**Table 5:** Diagnostic performance of the two radiologists using AI in Group 2 during the second reading.

	SE (%)	SP (%)	AUC	*P*-VALUE	OR [95% CI]	PPV (%)	NPV (%)
**SR + AI**	81.25	89.47	0.8536	<0.0001	36.8 [6.6–204.3]	92.86	73.91
**JR + AI**	56.25	78.95	0.6760	0.0182	4.8 [1.3–17.8]	81.82	51.72

**Figure 1:**
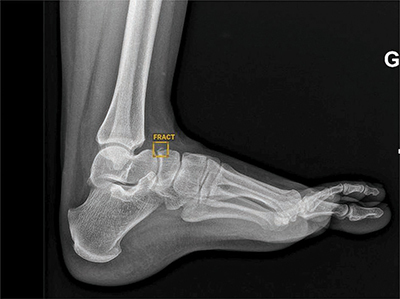
AI true positive response for fracture of navicular bone confirmed by the radiologist in charge of radiographic exams.

**Figure 2:**
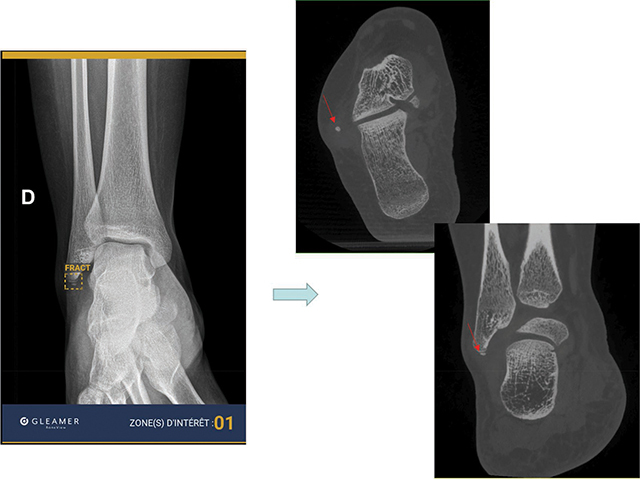
AI false positive response for fracture of the external malleolus, which CBCT confirmed to be a calcification.

**Figure 3:**
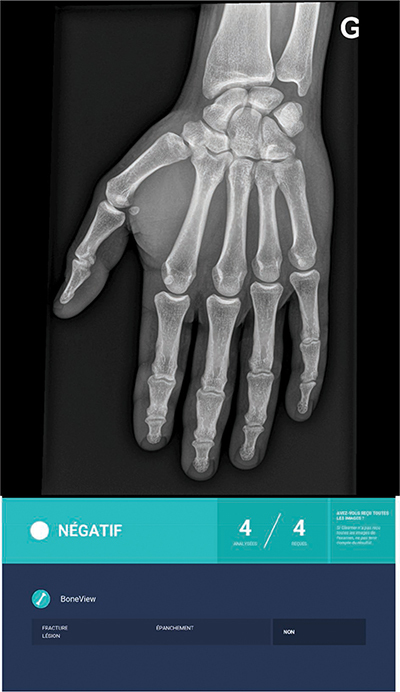
AI true negative response to a left-hand trauma.

**Figure 4:**
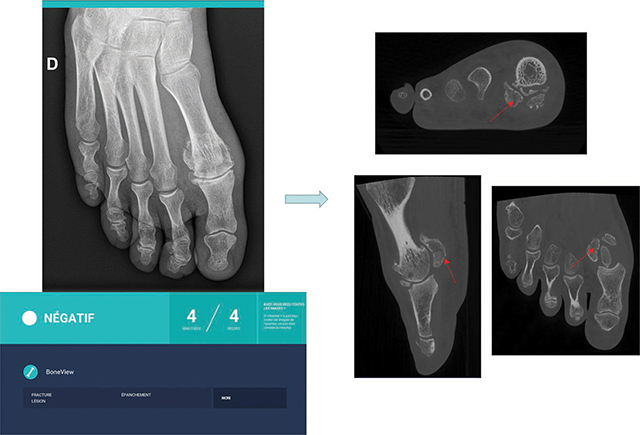
AI false negative response for a fracture of the lateral sesamoid, which CBCT confirmed.

Examples of true positives, false positives, and true and false negatives of AI are shown in Figures 1–4, respectively.

## Discussion

This study shows the following: (a) AI performance is close to that of an experienced MSK radiologist; (b) AI is even performing slightly better than this experienced radiologist for common fractures but underperforms for subtle traumatic lesions; (c) An unexperienced radiologist does not perform as well as AI, but AI helps him improving his performance.

Regarding AI performance, our results are fairly close to those of several studies in which the AUC varies between 0.85 and 0.95, slightly better than that of MSK radiologists (between 0.8 and 0.9) [3, 11, 18]. These performances confirm that the AI is excellent for detecting common post-traumatic fractures and has an excellent NPV, as shown in Figure 3. This can help a lot to improve young doctors’ skills and the management of fractures, even during night shifts when a radiologist is not always available. However, AI also proved to show false positives (such as old fractures, calcifications, and bone overlay), as illustrated in Figure 2. Radiologists have to keep this in mind when validating AI-positive results.

Moreover, as might be expected, the performances of AI were much lower for the detection of subtle fractures, as illustrated in Figure 4. In these particular cases, AI is far from perfect and may still stay behind the experienced radiologist’s performance. For sure, in complex fractures, even an experienced MSK radiologist may miss subtle lesions. This may, however, be mainly due to the radiographic technique itself that we compared to a high-resolution 3D technique, which is more effective [19]. But with this study, it is shown that the adjunction of AI to the experienced radiologist’s reading has the potential to increase the detection of subtle fractures and thus to reduce the number of additional 3D imaging studies. More data should be collected to confirm this finding.

Regarding radiology training, our results show that the JR benefits most from using AI, as its performance showed a much higher increase between first and second readings as compared to that of the MSK experienced radiologist. AI is thus a potential excellent teaching tool, if used as a second reading as recommended in the literature [20, 21] and as used in this study. Although, adding AI in a clinical setting might cause a lack of motivation for new radiologists to improve their skills for fracture reading and an excessive trust in AI without any medical reflection.

### Study limitations

First, the sample size for the two groups was not that large, and only performed in adults. Second, the radiologists’ performance was assessed in the absence of clinical information, which may have induced limited accuracy. Third, the number of readers was small, whereas some variations in reading performance could be observed among both experienced and inexperienced radiologists. Fourth, AI results were considered as positive even if one of the multiple fractures was not detected by AI. Thus, AI performance could be lower than presented. Fifth, there is a potential selection bias for the radiologists during the reading sessions, as they knew that radiographs were taken in a trauma setting with low velocity. Finally, magnetic resonance imaging (MRI) examination, or bone scintigraphy, should have been used as the reference method, as these two techniques are more effective than radiography and CBCT in detecting fractures.

## Conclusion

AI has demonstrated excellent reliability in detecting bone fractures on radiography and promises to be an indispensable learning tool in the training of junior radiologists. This technology is less useful for senior radiologists, proving that in detecting subtle fractures, the thinking of a human expert is not (yet) matched by that of AI. Further investigations should be carried out to improve the performance of AI software in these circumstances.
